# Design considerations for the enhancement of human color vision by breaking binocular redundancy

**DOI:** 10.1038/s41598-018-30403-y

**Published:** 2018-08-10

**Authors:** Bradley S. Gundlach, Michel Frising, Alireza Shahsafi, Gregory Vershbow, Chenghao Wan, Jad Salman, Bas Rokers, Laurent Lessard, Mikhail A. Kats

**Affiliations:** 1 Department of Electrical and Computer Engineering, University of Wisconsin-Madison, Madison, WI USA; 2 Department of Mechanical and Process Engineering, ETH Zurich, Zurich, Switzerland; 3 Department of Art, University of Wisconsin-Madison, Madison, WI USA; 4 Department of Materials Science and Engineering, University of Wisconsin-Madison, Madison, WI USA; 5 Department of Psychology, University of Wisconsin-Madison, Madison, WI USA; 6 McPherson Eye Research Institute, University of Wisconsin-Madison, Madison, WI USA; 7 Wisconsin Institute for Discovery, Madison, WI USA

**Keywords:** Colour vision, Imaging and sensing

## Abstract

To see color, the human visual system combines the response of three types of cone cells in the retina—a compressive process that discards a significant amount of spectral information. Here, we present designs based on thin-film optical filters with the goal of enhancing human color vision by breaking its inherent binocular redundancy, providing different spectral content to each eye. We fabricated a set of optical filters that “splits” the response of the short-wavelength cone between the two eyes in individuals with typical trichromatic vision, simulating the presence of approximately four distinct cone types. Such an increase in the number of effective cone types can reduce the prevalence of metamers—pairs of distinct spectra that resolve to the same tristimulus values. This technique may result in an enhancement of spectral perception, with applications ranging from camouflage detection and anti-counterfeiting to new types of artwork and data visualization.

## Introduction

**Figure 1:**
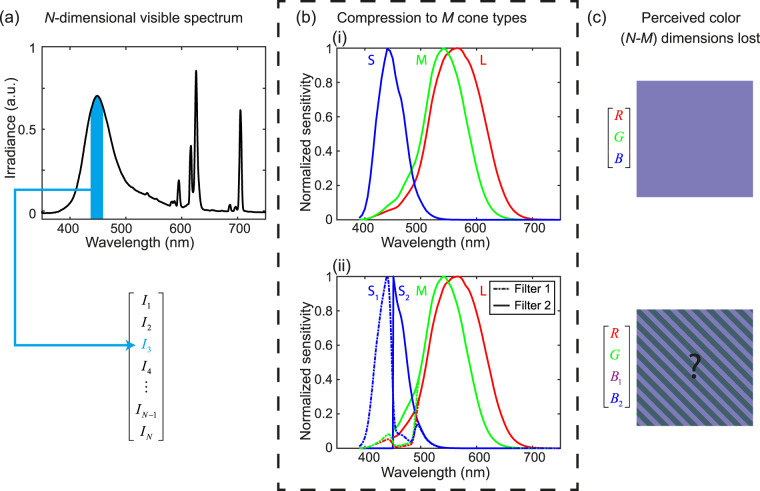
Compression of spectral information. (**a**) A sample spectrum generated by a cathode ray tube (CRT) monitor displaying a purple color. The filled rectangle represents a single spectral bin, if the continuous spectrum is divided into *N* bins. (**b**) (i) Normalized spectral sensitivity of the cone types for a typical trichromatic observer (*M* = 3). (ii) Normalized spectral sensitivity of the effective cone types for a typical trichromatic observer enhanced using our device ($M\cong 4)$. (**c**) A representation of the perceived color of the spectrum in (a). The case with $M\cong 4$ cannot be displayed, but extra spectral information would be present compared to the *M* = 3 case.

In the typical human eye, the three cone types—labeled “S” for short wavelengths, “M” for medium, and “L” for long—are sensitive primarily to light with wavelengths in the 390–530 nm, 400–670 nm, and 400–700 nm bands, respectively^1–4^. When excited by light, the signal from the cones is relayed though retinal ganglion cells, to the optic nerve, and then the brain, where it is further processed to produce a color sensation^5,6^. This process can be understood as a type of lossy compression from an *N*-dimensional spectrum, where *N* is the number of wavelength bins necessary to sufficiently approximate a continuous spectrum, into a color, which is a three-dimensional object (Fig. 1). A manifestation of this *N*-to-three compression is metamerism, a phenomenon in which different spectra resolve to the same tristimulus values (*i*.*e*., they appear as the same color, neglecting possible contextual effects)^2^. The number of cone types and the widths and separations of their spectral sensitivities govern the degree to which metamerism is a limitation of the visual system; for example, a hypothetical increase in the number of distinct cone types should result in a decrease in the prevalence of metamers. Cast in a signal processing perspective, an increase in the sampling rate of a spectrum (*i*.*e*., the number of distinct cone types) improves the ability to detect sharp features in the spectrum^7^.

Several studies have reported that a small percentage of humans, primarily women, express a mutated L cone in addition to the standard one, resulting in a total of four cone types, which may in principle enable vision with four color dimensions (tetrachromacy)^8–10^. Reports suggest that a few of these individuals can utilize this fourth photopigment type, and thus “perceive significantly more chromatic appearances” compared to typical, healthy humans with three cone types (trichromats)^11,12^. More broadly, it is reasonable to infer that an additional cone type would enhance spectral perception, provided subsequent neural processing can capitalize on its presence.

**Figure 2:**
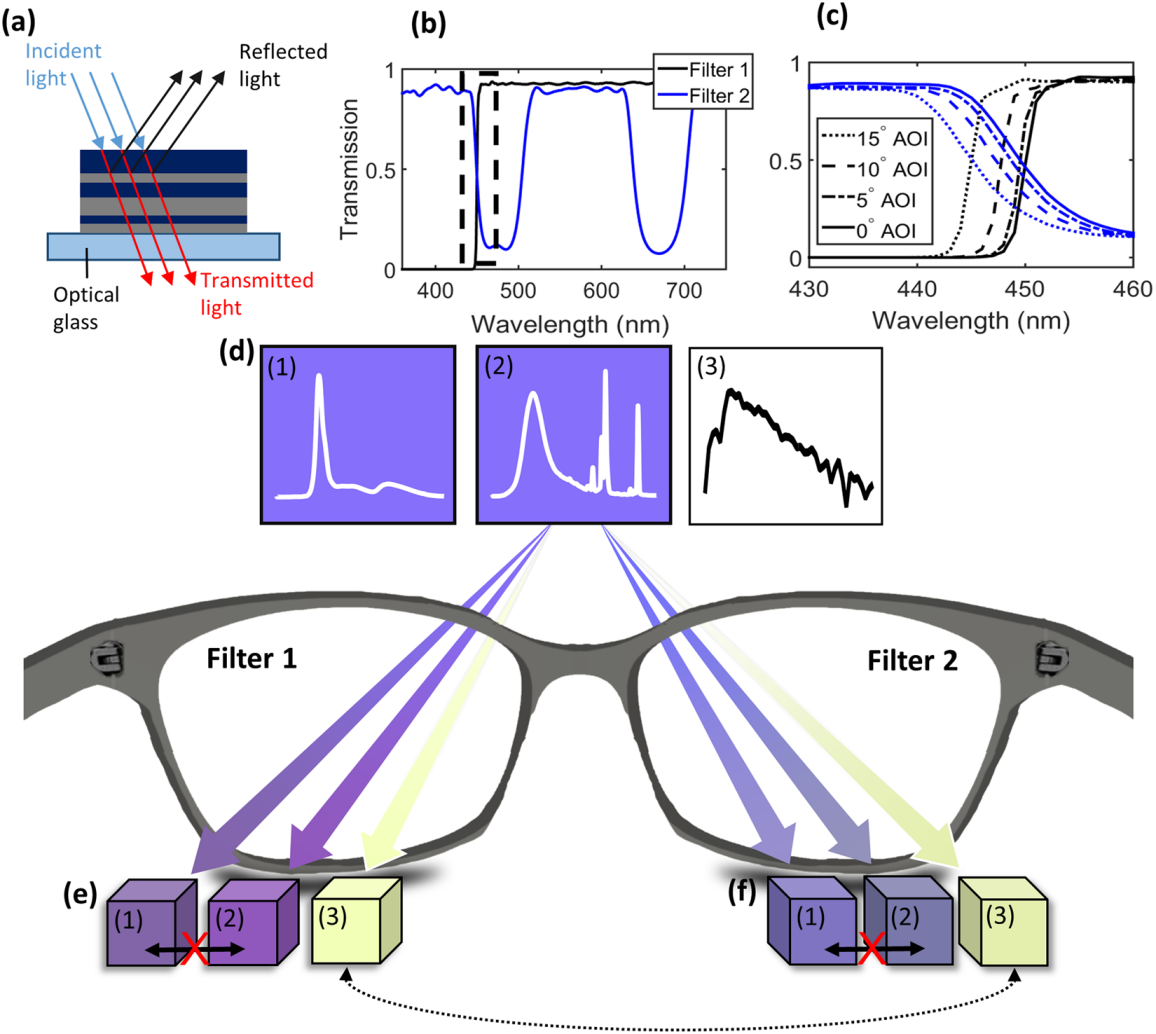
Wearable passive multispectral device comprising two distinct transmission filters. (**a**) Simplified schematic of an optical filter comprising several thin-film layers. (**b**) Measured transmission spectra of fabricated Filters 1 (black) and 2 (blue). (**c**) Magnified portion of the transmission spectrum from (b) including angle dependence with angle of incidence (AOI) from 0 to 15°. (**d**) Colors of an example metameric pair (1, 2) and D65 broadband white light (3), as perceived by a typical trichromat. The traces in each box are the underlying unfiltered spectra (arbitrary units), taken from our experiments, as described in Fig. 3. (**e**,**f**) Rendered monocular colors of the spectra in (d) after passing through Filter 1 and Filter 2, respectively. Note that e(1) and e(2), f(1) and f(2) are substantially distinct, while e(3) and f(3) are similar in color due to the white-balance constraint enforced in our design.

In this paper, we explore designs based on thin-film optical filters that may simulate tetrachromatic (and possibly higher-dimensional) color vision in typical trichromatic humans by increasing the number of *effective* cone types in the visual system comprising the two eyes and a passive optical device. The term “effective” is used here to differentiate between true tetrachromatic vision, which would be defined by four distinct retinal photopigments that generate distinct neural responses. This approach breaks the binocular redundancy of the two eyes, where the visual fields of each eye are overlapping, providing different spectral content to each eye via a wearable passive multispectral device comprising two optical transmission filters (Fig. 2).

A number of existing vision-assistive devices or techniques operate by breaking binocular redundancy, though usually in the spatial rather than spectral domain. Examples include hemianopia (partial blindness in the left or right visual field) treatment using spectacles with a monocular sector prism that selectively relocates the visual field in one eye, leaving the other eye unaffected, and thus conferring an additional 20° of visual-field sensitivity for binocular vision^13,14^, and the treatment of presbyopia by correcting one eye for near vision and the other for distance vision^15^.

We break binocular redundancy spectrally by using filters that selectively attenuate different wavelength bands to yield effective cone sensitivities (*i*.*e*., the products of the cone sensitivities and the filter transmission spectra) that are different between the two eyes. This approach is intended to increase the number of effective cone types while preserving most spatial information. In this vein, the use of two simple band-pass filters was previously demonstrated to increase the dimensionality of color vision in dichromatic individuals (*i*.*e*., those with two functioning cone types)^16^. Conversely, the goal in the present work is to enhance the dimensionality of a trichromat’s visual system to beyond that of a typical human. Such an approach was briefly suggested by Cornsweet in 1970^17^, but to our knowledge no specific design has been proposed or realized. We note that the use of even a single filter positioned in front of both eyes can help distinguish certain metamers^17^, with the caveat that previously distinguishable spectra can become metamers when viewed through the filter; that is, a similar number of metamers (usually more) are created as are destroyed. In contrast, the use of two filters might be used to decrease the overall number of possible metamers. For this work, if at least one of the two filters can be used to differentiate a pair of spectra, we consider the pair to no longer be metamers. We emphasize that no behavioral data is presented in this manuscript.

## Results and Discussion

### Filter Design and Construction

The filter pair was designed using a standard psychophysical model to determine the perceived (monocular) colors corresponding to particular spectra^2,18^. The perceived colors were calculated using the International Commission on Illumination (CIE) 1931 2° standard-observer matching functions, and monocular color differences (*e*.*g*., between colors 1 and 2) were calculated in the CIELAB color space using a standard color-difference metric (see *Methods* for further details)^2,19^:

1 $${\rm{\Delta }}{E}_{12}=\sqrt{{({L}_{2}-{L}_{1})}^{2}+{({a}_{2}-{a}_{1})}^{2}+{({b}_{2}-{b}_{1})}^{2}}$$

The filters were designed to enhance the ability of a typical trichromatic viewer to discriminate spectra while limiting adverse effects. For simplicity, we focused on a design that splits the response of the S cone, thus transforming the trichromatic visual system into one that simulates tetrachromatic vision. The S cone was chosen because its responsivity has relatively little overlap with those of the M and L cones (Fig. 1b(i)), so it can be attenuated while minimizing the impact on the effective responsivity (*i*.*e*., the product of cone responsivity and the filter transmission response) of the other two cone types. To provide approximate parity between eyes, we partitioned the S cone responsivity such that each eye retains approximately half of the original response spectrum (Fig. 1b(ii)). Our secondary design goal was to ensure that the transmission of broadband white light (defined using CIE illuminant D65)^20^ through the two filters results in similar tristimulus values. This constraint was put in place to minimize potential baseline disparities (*e*.*g*., when viewing broadband white objects) between the eyes when the device is used in daylight. Though a particular implementation of this type of filter-based device generally depends on the illuminant chosen, the design presented here should work well for most illuminants along the Planckian locus^21^.

The final device presented here comprises a 450 nm long-pass filter (Filter 1) and a 450–500 nm, 630–680 nm double-band-stop filter (Filter 2) (Fig. 2b,c); the filter designs were optimized by varying their stopband/passband positions and transmittances using simulated annealing to minimize the CIE ΔE color difference of D65 white light passing through Filters 1 and 2 (See *Methods* for further details). The 450 nm transition between Filters 1 and 2 is at the peak sensitivity of the S cone, and partitions it in half. However, due to the non-zero sensitivity of the M and L cones in the 450–500 nm region [Fig. 1b(i)], the M and L cones are also (unintentionally) attenuated by Filter 2. A second stopband at 630–680 nm was introduced to attenuate the effective responsivity of the M and L cones to broadband white light to preserve color balance. Though the filter designs were optimized for these constraints, we note that the design presented here is a proof of concept, and is not a unique or globally optimal solution.

To reduce cost and manufacturing time, an off-the-shelf component (450LP RapidEdge, Omega Optical), was used for Filter 1. The optimized transmission function of Filter 2 was realized using conventional thin-film technology^22^, with alternating layers of silicon oxide (SiO_2_, *n* = 1.46) and tantalum oxide (Ta_2_O_5_, *n* = 2.15) (Fig. 2a,b), deposited on an NBK7 glass substrate (see *Methods*). The two filters were then characterized by angle-dependent transmission spectroscopy, demonstrating that the transmission spectra are robust to incidence angles up to 5° away from the normal (Fig. 2c). Following fabrication, the filters were constructed into a pair of glasses.

### Experiments

**Figure 3:**
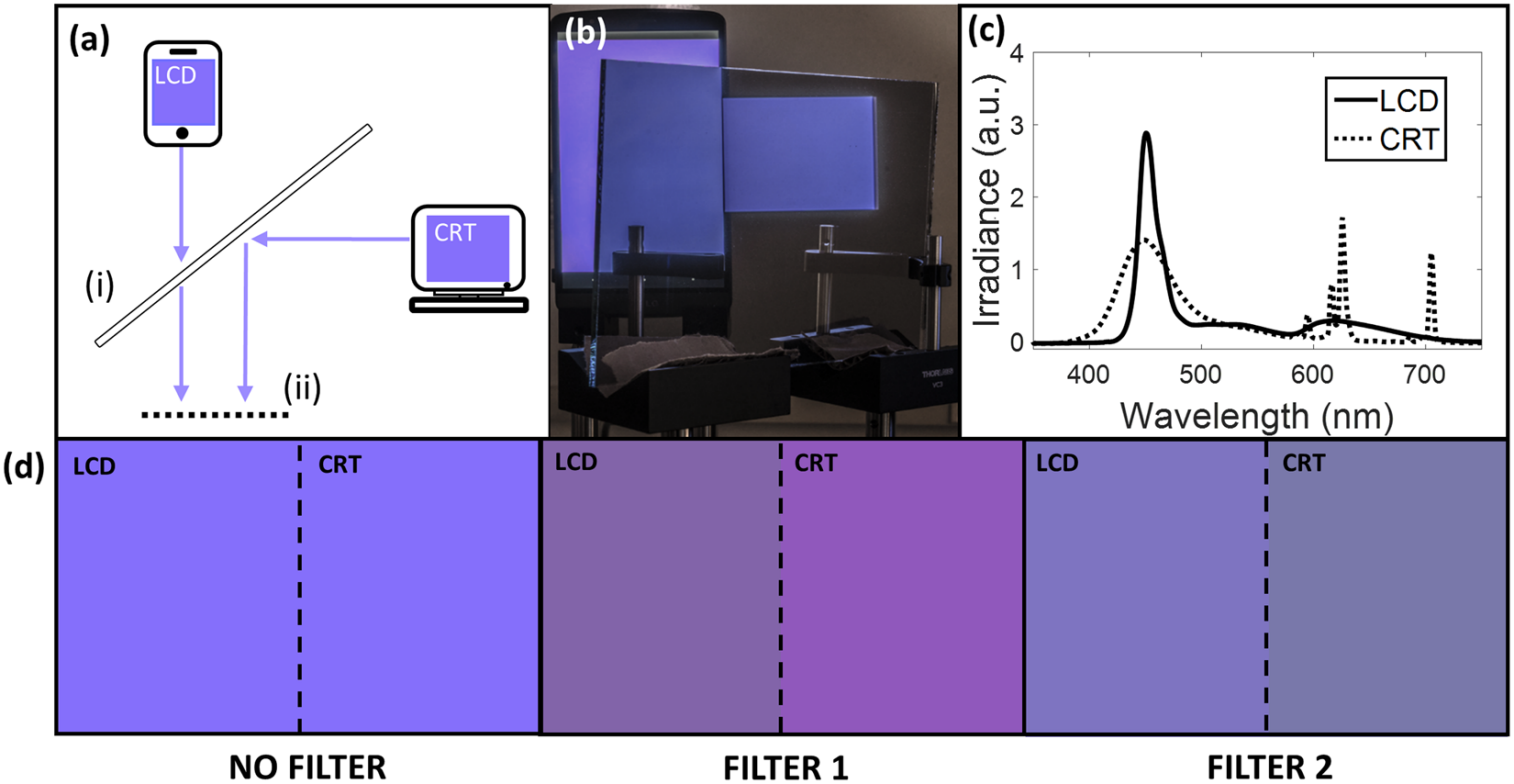
Splitting metamers by breaking binocular redundancy. (**a**) Schematic of our metamer generation setup. Images from two monitors, an LCD and a CRT display, are combined using a 50/50 beam splitter (i), to be viewed at location (ii). The two monitors use different emission mechanisms, and thus generate different spectra for the same color. (**b**) Photograph taken at position (ii) in the schematic. (**c**) Measured emission spectra from the LCD (solid) and CRT (dashed) monitors while displaying the same purple color. Spectra are shown with arbitrary units. (**d**) Rendered colors of the spectra in (c) viewed through no filter, Filter 1, and Filter 2, respectively, showing that the metamers can be distinguished using either filter. See the supplementary information for discussion of the difference between rendered colors in (d) and those in the photograph in (b).

To test the performance of this design, we constructed a setup that generates metameric spectra using a liquid crystal display (LCD, True HD-IPS display on LG G3 smartphone) and a cathode ray tube (CRT, Dell E770P) monitor (Fig. 3a,b). The displays use different emission mechanisms, and thus produce distinct spectra when displaying the same color (See *Supplementary Information* for further analysis)^23,24^. Blocks of color generated by the displays were presented side by side using a 50/50 beam splitter, and the colors were individually adjusted until no perceivable color difference was present. The emission spectra of each monitor were recorded using a free-space spectrometer, allowing for chromaticity and color-difference calculations to be made given a standard observer. A threshold value of 2.3 for the CIE Δ*E* “just noticeable difference” was taken to define perceptually indistinguishable spectra (*i*.*e*., Δ*E* < 2.3)^25^. See *Methods* for further details of the experimental setup.

One representative example from this dual-display setup, using a pair of metamers that appear purple, is shown in Fig. 3b–d. Without the use of either filter, the two different spectra appeared as identical patches of color. However, when observed through either of the filters, the two can be differentiated. Subjectively, we observed that, by looking at a particular patch through both filters simultaneously (*i*.*e*., Filter 1 over the left eye, Filter 2 over the right), a color percept is observed that is different from the color perceived through either filter individually or with no filter. We note that a related study involving dichromats demonstrated an increase in color dimensionality using band-pass filters, which the authors suggest the effect could be related to binocular lustre^16^. Our proposed capability to distinguish metamers by breaking binocular redundancy may be affected by binocular lustre and/or rivalry, which might be advantageous provided it can be used as a cue to a difference in hue. For example, a recent report has demonstrated that compression artifacts of pixels in virtual reality images can be easily detected due to lustre^26^. Note that lustre and rivalry are both dynamic phenomena, even for static stimuli^27–29^; thus the use of lustre/rivalry may result in a tradeoff between temporal and spectral resolution. Though our current experiments do not directly investigate the effect of lustre or rivalry, or probe to what degree the neuronal processing system of a trichromat can take advantage of the extra spectral information resulting from binocular filtering, this can be explored in future work.

We note that substantial differences in luminance between the two eyes, such as for spectra that transmit chiefly through only one filter, might lead to the Pulfrich effect^30^; however, our design was optimized to minimize differences in appearance of Illuminant D65, and the binocular luminance disparity required for the Pulfrich effect to occur is unlikely for most commonly occurring (*i*.*e*., smooth/broad) spectra.

### Calculation of metamer reduction

Broadly stated, the number of cone types and their frequency-dependent responsivities determines the extent to which metamerism is a limitation to the visual system. Our method is meant to increase the number of effective cone types, which should decrease the number of potential metamers, provided the subsequent neuronal processing can adapt appropriately (which seems to occur in the case of spatial multiplexing used for vision-assistive devices^13,14^). In general, quantitatively determining the decrease in the metamer frequency is difficult because the set of possible metamers is not bounded. Nevertheless, various metrics can be applied to roughly estimate this quantity. For this work, we developed two separate metrics that describe this decrease in metamer frequency given the following two conditions: *Condition* 1: Without the use of filters, a metamer pair is defined by two spectra with a color difference Δ*E* < 2.3^25^. *Condition* 2: With the use of binocular filters, such as those in Fig. 2b, a metamer pair is defined by two spectra with a monocular color difference Δ*E* < 2.3 in each eye. That is, a pair of spectra is a metamer *if and only if* it is a metamer in each eye individually. We do not consider the possibility of other perceptual effects such as binocular rivalry or dichoptic color mixing.

**Figure 4:**
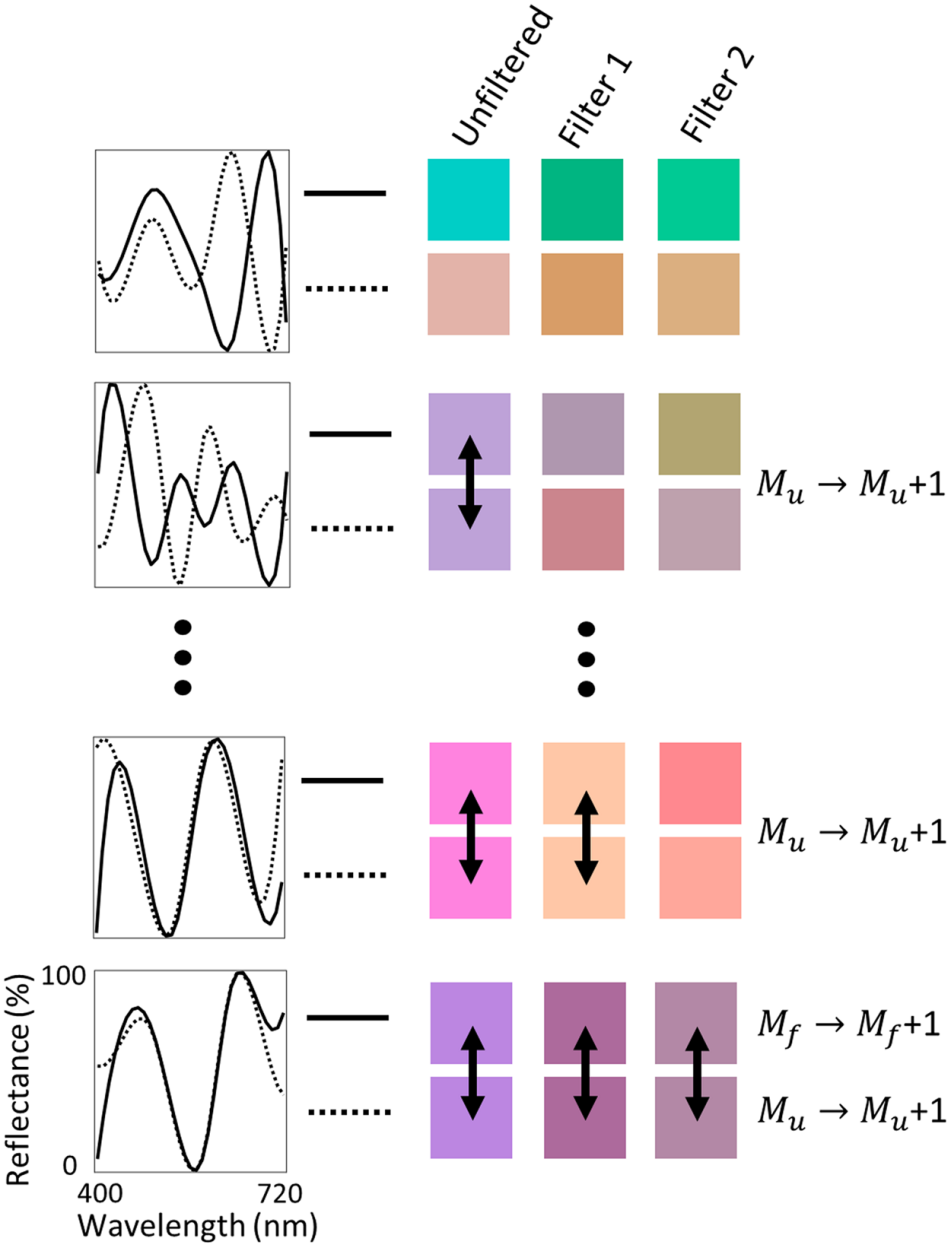
Graphic representation of the Monte-Carlo metamer-reduction calculation, where four pairs of randomly generated spectra are selected as an illustration. For the actual calculation, many pairs are generated. The corresponding rendered colors when viewed under illuminant D65 are shown for unfiltered, filter 1, and filter 2 cases, respectively. If a metamer is present in the unfiltered case, *M*_*u*_ is incremented by 1. If metamers are present when viewed through *both* Filter 1 and Filter 2, *M*_*f*_ is incremented by 1. Note that if a metamer exists when viewing through Filter 1 but not Filter 2, or vice versa, the spectra are considered distinguishable and *M*_*f*_ is not incremented.

Our first metric uses a Monte Carlo simulation to probe the effect binocular filters have on the perception of pairs of spectra, given the conditions above (Fig. 4). To start, a pair of reflectance spectra is generated by stochastically sampling intensity values from a uniform distribution at regularly spaced intervals within the visible wavelength range (*i*.*e*., at ${\lambda }_{1},{\lambda }_{2\ldots }{\lambda }_{{N}_{s}-1},{\lambda }_{{N}_{s}}$, where *N*_*s*_ is the total number of sampling points). The sharpness of the reflectance spectra was adjusted by changing *N*_*s*_, with larger numbers leading to sharper features, and were interpolated at 10 nm intervals using a cubic spline to create smooth spectra. We assumed illuminant D65, and then filtered the reflected spectra by the filter transmission responses given in Fig. 2b. ΔE color differences were calculated between the pairs of spectra for the unfiltered case, and through Filter 1 and Filter 2. The method was performed for various number of iterations (*N*_*i*_), which varied from 1,000 to 20,000,000, and the number of unfiltered (*M*_*u*_) and filtered (*M*_*f*_) metamers were recorded for each trial. We then defined a metric that represents the decrease in metamer frequency upon filtering:

$${P}_{m}=\frac{{M}_{u}}{{M}_{f}}$$

For example, *P*_*m*_ = 2 represents a two-fold decrease in the number of metamers using the two filters. The results from this simulation, for several sampling values (*N*_*s*_) and iteration numbers (*N*_*i*_), are given in Fig. S5 of the *Supplementary Information*. Given the simulation conditions, the filters in this work result in up to a ~15× decrease in the number of metamers for randomly generated spectra; this effect appears to be greatest for moderately sharp spectral features (*N*_*s*_ = 15), and drops off for very broad or very sharp spectra. We note that, though the given metric seems to converge for larger iteration numbers (See *Methods* and *Supplementary Information*), these measures are only meaningful to within a factor of ~2 due to the stochastic nature of this calculation.

As further verification of the apparent decrease in metamer frequency, we also developed a more-abstract method (See *Supplementary Information* for a complete description of this calculation, abridged here for clarity). Rather than comparing stochastically generated spectra, as above, this method aims to calculate the overall number of spectra that map to perceptually indistinguishable tristimulus values (Δ*E* < 2.3). For a given reference point in LAB space, [*L*_*o*_, *a*_*o*_, *b*_*o*_], the number of metamers (with respect to the reference point) was determined by counting the spectra, *I*(*λ*), that map to LAB coordinates within a sphere of radius 2.3 around the reference point. We determined the number of metamers by calculating the volume of spectra, represented by an ellipsoid in *N*-dimensional space, where *N* is the number of discrete wavelength bins that define a spectrum. However, calculating the exact volume of high dimensional ellipsoids in this case is difficult; instead, we calculate the volume of the max-inscribed ellipsoid subject to box constraints, which represents an upper bound of the true value^31^ (see *Supplementary Information* for more details). The volume of this ellipsoid represents the number of metamers, for a given reference point, for the unfiltered case (*V*_*u*_). For the filtered case, the union of two ellipsoids, corresponding to each filter individually, represents the number of metamers (*V*_*f*_); this is equivalent to *Condition* 2 above, where we assume that monocular metamerism must be present in both eyes simultaneously to yield indistinguishable color percepts in the filtered case. Thus, the overall decrease in metamer frequency is given by:

$${F}_{m}=\frac{{V}_{u}}{{V}_{f}}$$

where *F*_*m*_ = 2, as an example, represents a two-fold decrease in metamer frequency. This process was repeated for 500 LAB reference points from randomly generated spectra to adequately sample the color space. The number of wavelength samples (*N*_*s*_) was also varied to again explore the effect of spectral sharpness; as in the Monte-Carlo simulation, the decrease in metamer frequency occurs around 12–16 bins. Using this metric, we estimate a decrease in metamer frequency by one-to-two orders of magnitude when using our thin-film filter pair (see Table S1 in the *Supplementary Information*). By the same metric, a single-filter system designed to improve vision in color-vision-deficient individuals^32^ seems to provide no decrease in the frequency of apparent metamers.

## Conclusion

By breaking the inherent chromatic redundancy in binocular vision, our method aims to provide the user with more spectral information than is otherwise available. In the present design, the S cone is partitioned using a pair of filters that results in photoreceptor responses consistent with a visual system that utilizes approximately four cone types (*i*.*e*., simulated tetrachromacy). The S cone is more sensitive to blue-colored objects, and this approach can, *e*.*g*., be used for differentiating structural color versus natural pigments (See *Supplementary Information*)^33^. It is also possible to use similar methods to design filters that more strongly affect metamers that appears as green and red, that are more prevalent in nature^34^. While the possibility of natural tetrachromacy in a fraction of the population has received both academic and popular interest^10–12^, the technology demonstrated here has the potential to simulate tetrachromatic vision in anyone with typical, healthy trichromatic vision. The extent to which observers can (or can learn) to take advantage of the additional spectral information is yet to be determined, and requires behavioral/perception studies.

Given two eyes and three types of cones, it should be possible to increase the number of effective cones up to six using our approach, and potentially even more with spatial or temporal multiplexing. It may also be possible to generate personalized designs to improve color discrimination for individuals with color-vision deficiencies. This technology can be integrated in a simple pair of eyeglasses or sunglasses, and could have immediate applications in camouflage detection, quality control, anti-counterfeiting, and more. More broadly, the ability to see many more colors has intriguing opportunities for design and artwork, and for data representation with extra color channels.

## Methods

### Color calculations and CIE color differences

The International Commission on Illumination (CIE) standard was used for color calculations, represented by the equation^2,18^:

$${\rm{\Theta }}={\int }_{{\lambda }_{1}}^{{\lambda }_{2}}\,\bar{\theta }(\lambda )T(\lambda )I(\lambda )d\lambda /{\int }_{{\lambda }_{1}}^{{\lambda }_{2}}\,\bar{y}(\lambda )I(\lambda )d\lambda $$

where Θ = [X; Y; Z] are the XYZ tristimulus values, $\bar{\theta }(\lambda )=[\bar{x}(\lambda ),\bar{y}(\lambda ),\,\bar{z}(\lambda )]$ are the 1931 CIE 2° standard observer matching functions, *T*(*λ*) is the transmission spectrum of the filter, and *I*(*λ*) is the spectral irradiance of light passing through the filter. The XYZ tristimulus values can be transformed to a different color space (e.g., RGB); here, we use the CIELAB color space because it is more perceptually uniform and allows for straightforward calculations of perceived color differences. The XYZ to LAB transformation is given by^19^:

$${L}_{1}=116f(\frac{Y}{{Y}_{n}})-16,\,{a}_{1}=500[f(\frac{X}{{X}_{n}})-f(\frac{Y}{{Y}_{n}})],\,{b}_{1}=200[f(\frac{Y}{{Y}_{n}})-f(\frac{Z}{{Z}_{n}})],$$

where

$$f(t)=\{\begin{array}{ll}{t}^{1/3}, & t > {(\frac{6}{29})}^{3}\\ \frac{1}{3}{(\frac{6}{29})}^{2}t+\frac{4}{29}, & otherwise\end{array}$$

and *X*_*n*_, *Y*_*n*_, *Z*_*n*_ are the tristimulus values of the reference white point. Here, white light is defined by the CIE D65 standard illuminant, which roughly corresponds to average mid-day solar illuminance. The white point of D65 is (95.047, 100.000, 108.883)^20^.

### Filter Design

An iterative optimization approach was used to design the transmission of Filter 1 and Filter 2, where the filter responses became more complex as our intuition grew between iterations. This approach was used to meet the primary design goal, splitting the spectral response of the S cone between eyes, while also enforcing other optimization conditions such as a perceptual color balance for D65 white light between eyes.

The final filter designs comprise a 450 nm longpass filter (Filter 1) and a 450–500 nm, 630–680 nm double bandstop filter (Filter 2). The 450 nm transition region, where Filter 1 cuts on and the first bandstop of Filter 2 cuts off, occurs roughly at the peak sensitivity of the S cone. Therefore, Filter 1 transmits the long wavelength half of the S cone, while the first stopband of Filter 2 transmits the short wavelength half of the S cone. However, due to the nonzero sensitivity of the M and L cones between 450–500 nm, their sensitivities are also inadvertently attenuated, impacting the D65 color balance between eyes. The second bandstop of Filter 2, between 630–680 nm, attenuates the long wavelength tails of the M and L cone sensitivities, restoring color balance between eyes. A 450 nm longpass filter (Omega Optical, 450LP RapidEdge) was chosen as Filter 1. Filter 2 was optimized using constrained optimization by linear approximation (COBYLA) to minimize the merit function: Δ*E*/*Width*_*bandstop*_, where Δ*E* is the color difference between Filter 1 and Filter 2 when transmitting D65 white light and *Width*_*bandstop*_ is the spectral width of the short-wavelength band-stop region of Filter 2. This merit function ensures satisfactory D65 color balance between eyes while also maximizing the difference between filters, enhancing their ability to distinguish spectra. For Filter 2, the transmittance of the pass and stop-bands were constrained between 5–15% and 80–95%, respectively, and the longer wavelength stopband was constrained between 600–700 nm to prevent attenuation of the M and L cones at their peak sensitivities (~550 nm, ~580 nm respectively). This procedure yielded an optimized response for Filter 2 with stopbands at 450–500 nm and 630–680 nm, and stopband/passband transmittance of 10% and 90%, respectively; the color difference for illuminant D65 between Filter 1 and Filter 2 is Δ*E* = 5.21, with chromaticities of (0.348,0.406) and (0.35,0.415), respectively.

### Thin-film optimization and construction

The required film thicknesses were determined by conventional thin-film optimization methods, including gradual evolution^35^ and needle optimization^36^, to implement the target transmission function. The final stack was constrained to be less than 75 total layers, and each layer between 10 and 500 nm thick. The filter was optimized such that the transmission would not change significantly for incident angles up to 5° away from the normal. The films were deposited using ion-assisted sputtering onto an NBK7 glass substrate at a thin-film foundry (Iridian Spectral Technologies, Ontario, Canada). See *Supplementary Information* for more information about the thin-film design.

### Metamer Generation

An LCD (True HD-IPS on LG G3 smartphone) and CRT (Dell E770P) display were used to generate metameric pairs. The monitors were placed at a 90° angle from one another, and a large 50/50 beam splitter (Edmund Optics) was placed at 45° between the displays such that images from the two displays could be projected directly next to each other with no border. To find a metamer, a block of color was displayed on the CRT display, and a user-controlled 3-axis joystick was used to adjust the LCD image until no perceivable color difference was detected by the observer; the 3-axis joystick controlled colors in the HSV color space. The entire experimental setup was enclosed in a wooden box, painted black on the inside, and square apertures were placed on each display to ensure the images were displayed with black backgrounds to mitigate possible contextual perception effects^28^. Spectra from each monitor were acquired using a free-space spectrometer (Ocean Optics FLAME VIS-NIR with cosine corrector), normal to and adjacent to each display screen. Spectra for the white point of each monitor are shown in the S*upplementary Information*.

### Monte Carlo Simulation

Reflectance spectra were calculated by generating random values at a defined number of sampling numbers (*N*_*s*_) within the visible wavelengths (400–720 nm) using Matlab’s rand function; *N*_*s*_ was varied between 4–35 points to define the sharpness of spectral features, and the spectra were interpolated using a cubic spline. CIE 1931 2° matching functions were used to calculate tristumlus values for illuminant D65 reflected from the objects. Illuminant D65 was used as the white-point for conversion to the CIELAB space. A threshold of Δ*E* < 2.3 was used to define indistinguishable tristimulus values^25^. The simulation was performed for several number of iterations (*N*_*i*_), from 1,000–20,000,000, to determine if the defined metric *P*_*m*_ converged for a given *N*_*s*_. For *N*_*i*_ greater than 1,000,000 values for *P*_*m*_ converged to values within ~20% of each other within a given *N*_*s*_.

### More-abstract calculation of metamer frequency

The volume approximation ratios were computed using CVX, a package for specifying and solving convex programs^37^. Reference points [*L*_0_, *a*_0_, *b*_0_] were chosen by uniformly sampling discretized spectra and mapping them to LAB tristimulus values. The computation was performed for various wavelength binning values (*N*_*b*_), between 7–18, to vary the broadness/sharpness of spectra, and 500 reference points were computed for each binning value. A detailed summary of this calculation, including a formalized mathematical treatment, can be found in the *Supplementary information*.

### Data Availability Statement

All data generated or analyzed during this study are included in this published article (and its Supplementary Information files).

## Electronic supplementary material

Supplementary Information
