# Author Correction: Design considerations for the enhancement of human color vision by breaking binocular redundancy

**DOI:** 10.1038/s41598-022-05068-3

**Published:** 2022-01-12

**Authors:** Bradley S. Gundlach, Michel Frising, Alireza Shahsafi, Gregory Vershbow, Chenghao Wan, Jad Salman, Bas Rokers, Laurent Lessard, Mikhail A. Kats

**Affiliations:** 1 Department of Electrical and Computer Engineering, University of Wisconsin-Madison, Madison, WI USA; 2 Department of Mechanical and Process Engineering, ETH Zurich, Zurich, Switzerland; 3 Department of Art, University of Wisconsin-Madison, Madison, WI USA; 4 Department of Materials Science and Engineering, University of Wisconsin-Madison, Madison, WI USA; 5 Department of Psychology, University of Wisconsin-Madison, Madison, WI USA; 6 McPherson Eye Research Institute, University of Wisconsin-Madison, Madison, WI USA; 7 Wisconsin Institute for Discovery, Madison, WI USA

Correction to: *Scientific Reports* https://doi.org/10.1038/s41598-018-30403-y, published online 10 August 2018

The Acknowledgements section in the original version of this Article is incomplete.

“B.G. is supported by the National Science Foundation Graduate Research Fellowship under Grant No. DGE-1256259. This work was supported by startup funds from UW Madison. We also thank Middleton Spectral Vision for access to their hyperspectral imaging systems.”

should read:

“B.G. is supported by the National Science Foundation Graduate Research Fellowship under Grant No. DGE-1256259. This work was partially supported by startup funds from UW Madison, and partially by the AFOSR (FA9550-18-1-0146). We also thank Middleton Spectral Vision for access to their hyperspectral imaging systems."

