# Supplementary material for: Design considerations for the enhancement of human color vision by breaking binocular redundancy
Source: Sci Rep. 2018 Aug 10;8:11971. doi: 10.1038/s41598-018-30403-y (PMC6086919; doi:10.1038/s41598-018-30403-y)
Supplement: Supplementary file 1 — Supplementary Information [file 41598_2018_30403_MOESM1_ESM.pdf]

Supplementary Information

Design considerations for the enhancement of human color vision by breaking binocular redundancy

Bradley S. Gundlach[1], Michel Frising[1,2], Alireza Shahsafi[1], Gregory Vershbow[3], Chenghao Wan[1,4], Jad Salman[1], Bas Rokers[5,6], Laurent Lessard[1], Mikhail A. Kats[1,4,6*]

[1]Department of Electrical and Computer Engineering, University of Wisconsin-Madison, Madison, WI
[2]Department of Mechanical and Process Engineering, ETH Zurich, Zurich, Switzerland
[3]Department of Art, University of Wisconsin-Madison, Madison, WI
[4]Department of Materials Science and Engineering, University of Wisconsin-Madison, Madison, WI
[5]Department of Psychology, University of Wisconsin-Madison, Madison, WI
[6]McPherson Eye Research Institute, University of Wisconsin-Madison, Madison, WI

Narrative explanation of the filter design process

The filters in this work were designed using a white-balance condition that ensures that broadband "white light" that passes through the two filters is perceived similarly, to prevent significant color clashing between the two eyes under typical viewing conditions. Simultaneously, the filters are designed to be sufficiently distinct, which results in each eye receiving different spectral information. We used a design approach where each revision increased in complexity, building intuition at each design stage.

For the first revision, a brick-wall longpass filter and a band-stop filter were used to split the blue cone response without significantly affecting the other cone types (Fig. S1). The filters were constrained such that the longpass filter cut-on wavelength was equal to the band-stop filter cut-off wavelength, which ensured that at least one eye was sensitive to every region of the visible wavelengths (*i.e.*, no wavelength was attenuated by both filters). The band-stop filter cut-on wavelength was chosen to be 450 nm to minimize the effect of the filters on the M and L cone responses; thus, we were left to decide the longpass cut-on and band-stop cut-off wavelengths (which are enforced to be equal), and which must be below 450nm. With these constraints, the position of the cut-on/cut-off wavelength was optimized in order to minimize the CIE ΔE color difference between D65 white light passing through each filter. Figure S1(a) shows the result of this optimization, with a minimum ΔE color difference of 0.596 with the cut-on/cut-off wavelength at 437 nm. A ΔE color difference less than 2.3 typically means that the colors are indistinguishable. The resulting optimized filters are: a longpass filter (Filter 1) with a 437 nm transition wavelength, and a band-stop filter (Filter 2) with a stopband of 437 - 450 nm. Figure S1(c) shows the rendered color of D65 white light through

each filter, and demonstrates the excellent white-balance between filters, because the color samples are indistinguishable. However, the band-stop width of the resulting Filter 2 is only 13 nm, quite low in comparison to the ~300 nm range of the visible wavelengths. In order to effectively differentiate spectral features in everyday scenes, which are typically quite broad (>10 - 20 nm), the band-stop width must be larger.

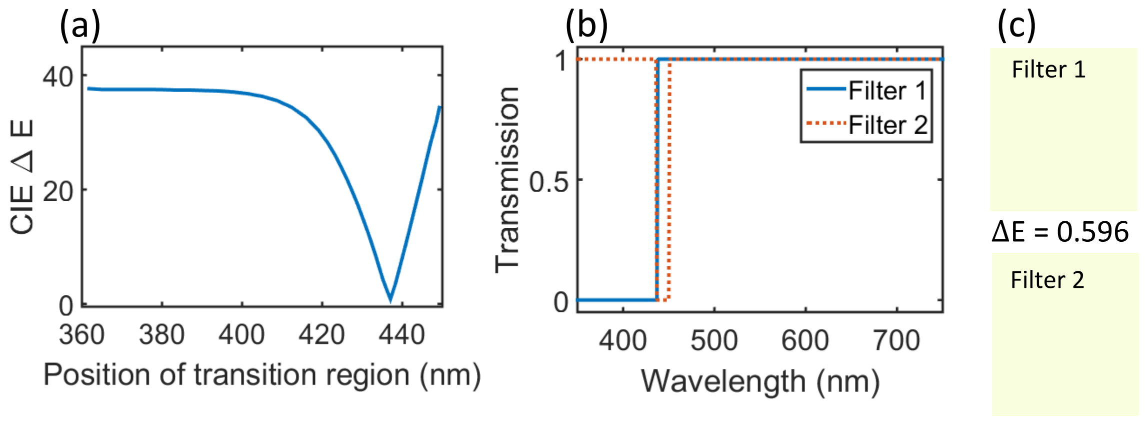

Figure S1: (a) Plot showing the CIE ΔE color difference between filters 1 and 2 when transmitting CIE D65 white light versus cut-on/cut-off wavelength. (b) Optimized transmission response for filters 1 and 2 after the first design revision, with a band-stop region between 437 – 450 nm. (c) Rendered colors for CIE D65 white light transmitted through each filter from (b), with an optimized ΔE color difference of 0.596.

Because this initial design produced a filter set with good white-balance, the same general approach was used in the following design revision. A similar optimization was performed as above, but the bandstop width of Filter 2 was constrained to be larger than 25 nm; this width was chosen such that it was larger than many typical spectral features found in nature, which could therefore be resolved with these filters. Instead of minimizing just the ΔE color difference between the filters, a modified merit function (MF) was used that also accounted for the filter width: $MF = \Delta E / Width_{bandstop}$. A brute-force optimization was performed, which varied the cut-on and cut-off wavelengths of the two filters (again with the longpass cut-on and bandstop cut-off wavelengths being equal). The transmission spectra of the optimized filters are given in Figure S2: a longpass filter (Filter 1) with a 450 nm cut-on wavelength, and a bandstop filter (Filter 2) with a stopband of 450 – 500 nm. The resulting filter set has a ΔE color difference of 14.15, significantly above the 2.3 just noticeable difference threshold. Therefore, it is clear that for the simple design using a longpass and single-bandstop filter, there is a tradeoff between white-balance and width of the band stop region.

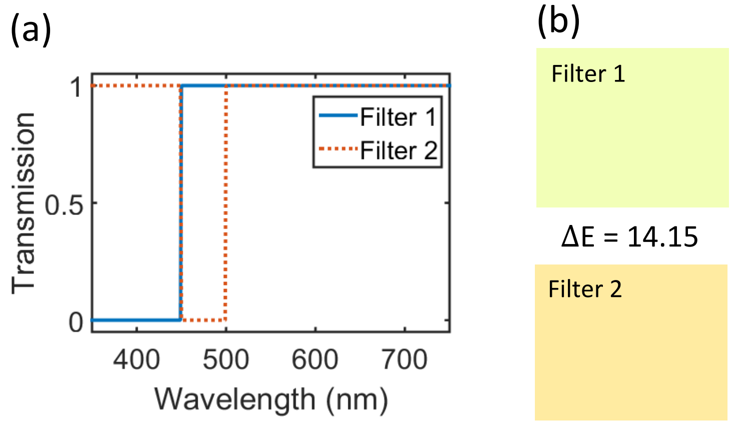

Figure S2: (a) Optimized transmission response for filters 1 and 2 after the second design revision, with a band-stop region of 450 - 500 nm. (b) Rendered colors for CIE D65 white light transmitted through each filter from (b), with an optimized ΔE color difference of 14.15

By considering the cone responses (Fig. 1c), it is clear why this filter design results in worse white balance. Beyond 450 nm, the longpass filter allows all wavelengths of visible light through; the M and L cones have very little response below 450 nm. However, the band-stop filter attenuates wavelengths between 450 and 500 nm, where the M and L cones have significant sensitivity. Therefore, the two filters affect the M and L cones very differently, which results in poor color balance. In particular, the band-stop filter (Filter 2) introduces a slight red tint compared to the longpass filter (Filter 1), which is also clear from the rendered colors in figure S2. Therefore, in order to maintain the high band-stop width of Filter 2 while improving the white balance of the filter pair, a second stopband was introduced to Filter 2 in order to soften the "red response" of the previous design. In this design, the shorter wavelength band-stop region of Filter 2 splits the S cone, whereas the longer wavelength band-stop region improves the white balance of the filter pair.

In the final design revision that was implemented and described in the main text, the response of the longpass filter (Filter 1) was not optimized any further; in fact, the transmission response of a commercially available 450 nm longpass filter was used to define Filter 1. This was done to decrease manufacturing costs, such that only Filter 2 required custom design and manufacturing. The short-wavelength band-stop region of the Filter 2 was also used from before (allowed to change only slightly during optimization), and a preliminary long wavelength band-stop region was added between 600 and 700 nm, to be optimized further. An error function was also implemented to smooth the transition regions; in the previous design revisions, the transition regions had a sharp vertical slope, which is difficult to achieve in practice. The smoothness of the filters can be adjusted by changing the proportionality constant (a) of the error function: $y = \mathrm{erf}(ax)$.

Unlike the previous revisions, Filter 2 was optimized using a more rigorous method compared to the brute force method used above. A constrained optimization by linear approximation (COBYLA) method was implemented in a stochastic basin-hopping algorithm to optimize the position of the cut-on/cut-off wavelengths, transmission of the pass and stop-bands, and slope of the transition regions. The transition regions of the filter's short-wavelength band-stop region was constrained within +/- 10 nm of their previous values; this was done to maintain the overall shape of the previous design while allowing some room for color balance optimization. The transmittance was constrained between 5 and 15% in the stopbands, and between 80 and 95% in the passbands, to allow for high throughput and relative ease of manufacturing. The error function proportionality constant was constrained between 0.25 and 1. The long-wavelength stopband region was constrained between 600 – 700 nm, but the band-stop width was not constrained; this was done to prevent attenuation of the M and L cones at their peak sensitivities (~550nm, ~580 nm respectively), while also preventing needless optimization beyond the visible wavelengths (> 700 nm). With these constraints in place, Filter 2 was optimized in order to minimize the modified merit function: $\Delta E/Width_{bandstop}$, where ΔE is the color difference between Filter 1 and Filter 2 when transmitting D65 white light and $Width_{bandstop}$ is the spectral width of the short-wavelength band-stop region of Filter 2. This procedure yielded an optimized response for Filter 2 with stopbands at 450 - 500 nm and 630 - 680 nm, and stopband/passband transmittance of 10% and 90%, respectively (Figure S3(a)). The rendered color of transmitted D65 white light through the filters is given in Figure S3(b), with a ΔE color difference of 5.21.

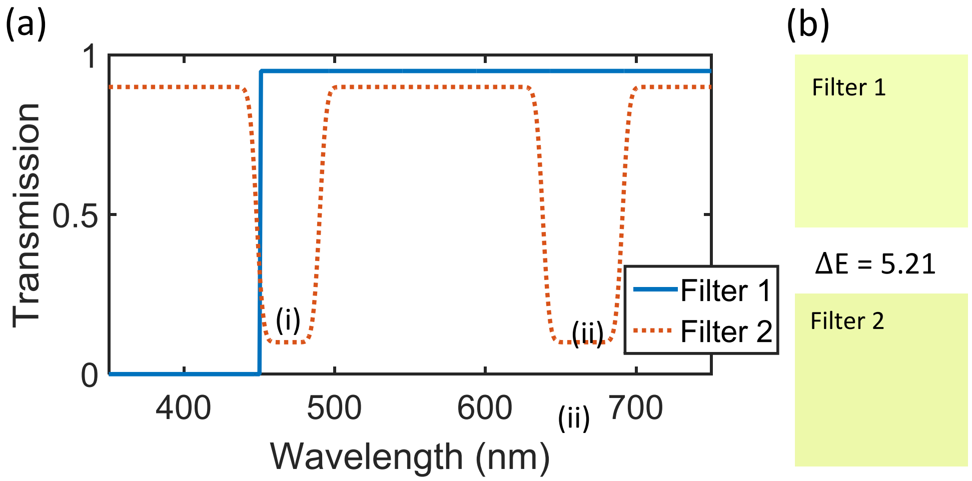

Figure S3: (a) Optimized transmission response for filters 1 and 2 after the third design revision, with band-stop regions between 450 – 500 nm (i) and 630 – 680 nm (ii). Region (i) splits the short-wavelength cone, region (ii) improves the white-balance of the filter pair (b) Rendered colors for CIE D65 white light transmitted through each filter from (a), with a ΔE color difference of 5.21.

Methodology for counting metamers in "more abstract" calculation:

In this section, we describe the technique by which we estimated the reduction of the occurrence of metamerism using our vision enhancement device.

Each spectrum $I(\lambda)$ is mapped to LAB tristimulus values $[L, a, b]$ via the CIE matching functions described earlier in the supplementary. Counting the number of metamers for a particular LAB reference point $[L_0, a_0, b_0]$ amounts to counting the number of different spectra $I(\lambda)$ that map to tristimulus values that are within a sphere in LAB space of radius ΔE of the reference point. The number of metameric spectra is infinite, so we instead compute a surrogate quantity. Roughly, we discretize each spectrum by wavelength so each spectrum can be abstracted as a point in a finite-dimensional space. We then count metamers by computing the volume that they occupy in this space. The details of the computation are described below.

1. Represent spectra by using N_S equally spaced samples in wavelength. For example, $I(\lambda)$ is represented as a vector $[I_1, I_2, \dots, I_{N_S}]$, which corresponds to the intensities at the wavelengths $[\lambda_1, \lambda_2, \dots, \lambda_{N_S}]$.
2. The map $\left[I_1, \dots, I_{N_S}\right] \rightarrow [L, a, b]$ from the discretized spectrum to LAB tristimulus values is smooth and nonlinear. Since the map is only being evaluated in a local neighborhood of the reference point, the map is well approximated by its first order Taylor expansion. This allows us to replace the nonlinear map with an affine function $g\left(I_1, \dots, I_{N_S}\right) = [L, a, b]$.
3. Let $S_0 = \{\, [L, a, b] \mid (L - L_0)^2 + (a - a_0)^2 + (b - b_0)^2 \leq \Delta E^2 \,\}$ be the set of tristimulus values indistinguishable from the reference point. The set of metameric spectra is given by the image of S_0 under the inverse map g^{-1}.
4. Since the inverse map g^{-1} is affine and the set S_0 is a sphere in LAB space, the image $g^{-1}(S_0)$ is an ellipsoid in the discretized spectrum space [S1]. Note that this ellipsoid is degenerate; it will be infinite in the directions corresponding to the kernel of g.
5. We assume that we are counting "reflection metamers" or "transmission metamers" under a certain illuminant. That is, we are excluding metamers generated by active emissive sources for the sake of this calculation, since including unbounded emissive sources complicates this calculation further. Under this assumption, the allowed intensities are not infinite, since every admissible spectrum has intensities bounded by the corresponding intensities of the illuminant. More formally, define the set of admissible spectra as $C_0 = \left\{ \left[I_1, \dots, I_{N_S}\right] \middle| \, 0 \leq I_k \leq I_k^{D65} \text{ for } k = 1, \dots, N_S \right\}$, where I_k^{D65} is the intensity at λ_k of the D65 illuminant.
6. The volume of metameric spectra is therefore the volume of the set $g^{-1}(S_0) \cap C_0$.

If the spectrum is filtered through a filter $T(\lambda)$, the map g must be replaced by a map g_T that accounts for the filter T. The derivation is otherwise identical; metameric spectra are given by the set $g_T^{-1}(S_0) \cap C_0$. If the spectrum is filtered through $T_1(\lambda)$ for one eye and $T_2(\lambda)$ for the other eye, spectra are only counted if they are metameric for *both* eyes. This results in the set $g_{T_1}^{-1}(S_0) \cap g_{T_2}^{-1}(S_0) \cap C_0$.

We can compare configurations (e.g. natural human vision versus vision augmented by our device, or vision augmented by two different filters sets) by comparing the volumes of their respective metameric spectra. For example, to compare the unfiltered case (natural human vision) to the case of vision modified by our two-filter passive multispectral device, we would compute the ratio:

$$\rho = \frac{\mathrm{Vol}(g^{-1}(S_0) \cap C_0)}{\mathrm{Vol}\left(g_{T_1}^{-1}(S_0) \cap g_{T_2}^{-1}(S_0) \cap C_0\right)}$$

A ratio of $\rho = 20$ would signify that metameric spectra are 20 times less abundant when the two-filter passive multispectral device is used as compared to the unfiltered case. Specifically, if spectra are sampled from a uniform distribution on intensities, a spectrum is 20 times less likely to be metameric.

Computing the ratio ρ is challenging because the volumes involved have irregular shapes; they are intersections of degenerate (high-dimensional) ellipsoids with box constraints. In order to approximate the ratio ρ, we approximate each volume by the volume of its max-volume inscribed ellipsoid. An illustration of a max-volume inscribed ellipsoid is shown below (Fig. S4).

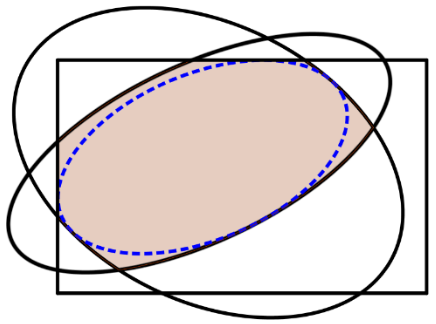

Figure S4: The shaded region is the intersection of ellipsoids and box constraints. The area is approximated by the max-area inscribed ellipsoid (dotted curve).

It turns out the max-volume inscribed ellipsoid can be efficiently computed using semidefinite programming techniques. See for example [S2]. We approximated the ratio ρ by using the inner ellipsoid approximation for both the numerator and denominator. Volume approximation ratios were computed using CVX, a package for specifying and solving convex programs [S3]. We computed approximate volume ratios for the two-filter case as well as the one-filter case, using the transmission spectrum of a filter sold by EnChroma for alleviating some of the adverse effects of red-green color vision deficiency (EnChroma filter [S4]). In each case, we tried several different discretization points N_S and we repeated the computation for 500 different reference points $[L_0, a_0, b_0]$. The reference points were selected by choosing discretized spectra at random, with reflectance values sampled from a uniform distribution between 0 and 1 (*i.e.*, N_S randomly sampled values per spectrum), and mapping them to LAB tristimulus values. A summary of the results is shown in Table S1. Using two filters results in a dramatic decrease in metamers, roughly consistent over the range of tested discretizations. In contrast, using a single filter (EnChroma in this case) has little effect on the number of metamers.

Discretization points (N_S)	7	9	12	14	16	18
Two filters, mean	22.977	115.823	120.580	160.634	178.020	111.243
Two filters, median	17.251	80.690	57.4080	85.885	77.153	41.369
EnChroma, mean	0.945	0.887	1.090	0.944	1.125	1.086
EnChroma, median	0.945	0.870	1.082	0.944	0.924	1.017

Table S1 – Results of the metamer ratio approximation. Mean and median ρ values are computed over 500 randomly generated spectra for each discretization. Using two filters reduces the frequency of metamers by a factor of about 50 on average, while using a single EnChroma filter has a negligible effect on the frequency of metamers

Monte Carlo Metamer Calculation

The Monte Carlo simulation, as described in the main text, was performed for several values of spectral sharpness N_s, with higher numbers signifying sharper features, and number of iterations N_i (Fig. S5). As discussed in the main text, P_m is largest for moderately sharp spectral features ($N_s = 15$). The metamer reduction metric seems to converge to within ~20% of neighboring values when N_i reaches ~1,000,000. Note there are missing values for low number of iterations because the sample size was not large enough to generate metameric spectra.

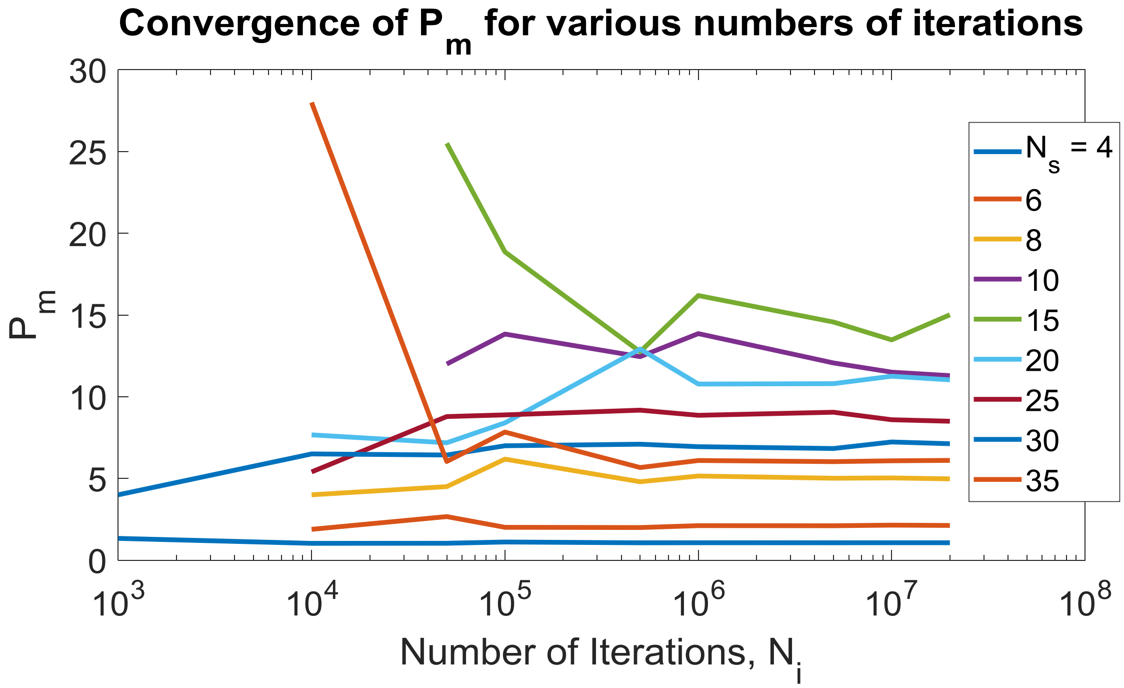

Figure S5: Convergence of the metamer reduction metric (P_m) as a function of N_s and number of iterations, N_i

Thin-film filter design

The filter response design goal (Fig. S3(a)) used in this work is realized by conventional thin-film design methods. A commercial thin-film design software (Optilayer) was used to optimize a two-material thin-film stack to adequately meet the design goal. Tantalum oxide (Ta_2O_5) was chosen as the high index (n = 2.15) material and silicon dioxide (SiO_2) was chosen as the low index (n = 1.46) material, as they are both easily deposited. The substrate was NBK7, a common optical glass. The final stack was constrained to be less than 75 total layers to keep costs down, and each layer between 10 – 500 nm to prevent stress cracks in thick films. Using these constraints in tandem with the provided filter design goal, the thin-film stack was optimized for incident angles between 0 - 10°. A representative stack design produced by Optilayer for the design of Filter 2 is given in Fig. S5.

The actual design for the device experimentally demonstrated in the main text was slightly modified from that of Fig. S6 by a thin-film foundry (Iridian Spectral Technologies, Ontario, Canada), though they did not share the precise thicknesses of the films with us due to their standard disclosure policy. Nevertheless, the specifics of the design are not critical as long as it implements the desired transmission spectrum (Fig. 2(b)).

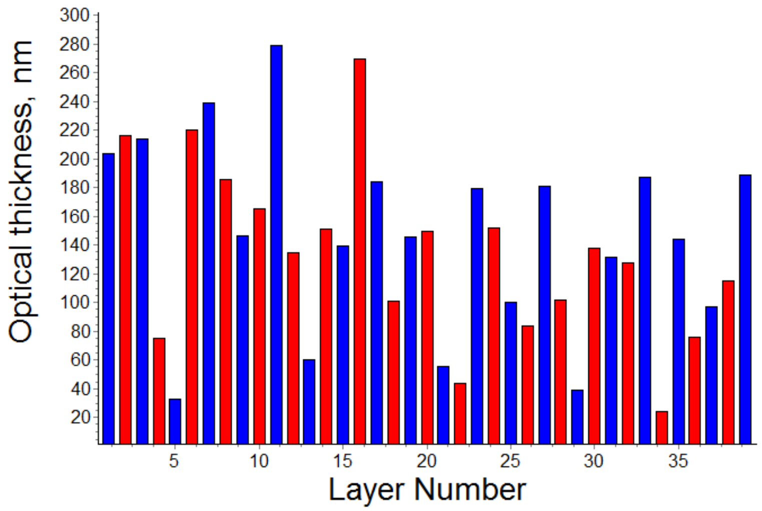

Figure S6: Thin-film filter stack design for filter 2 (Fig. S3(a)), using Ta_2O_5 (n = 2.15, blue) and SiO_2 (n = 1.46, red) dielectric layers.

Comparison of CRT and LCD monitors:

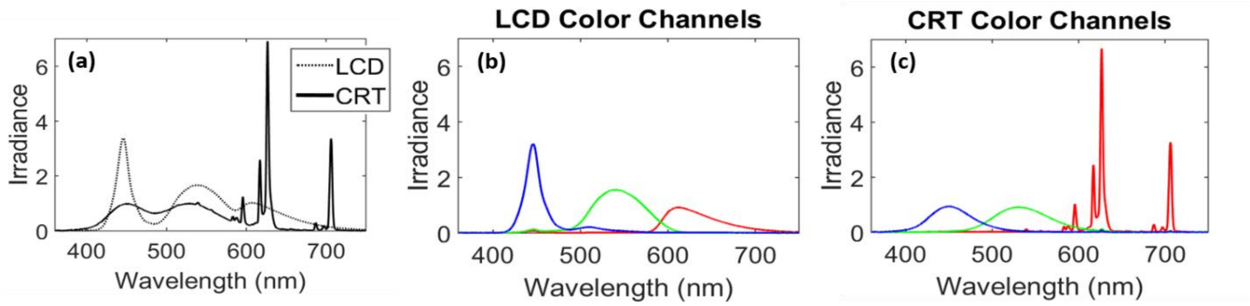

Figure S7: (a) Measured emission spectra of the LCD and CRT displays used in this work, displaying a white color (RGB = 255, 255, 255). (b, c) Measured emission spectra of each individual color channel (R, G, B) for the LCD and CRT display, respectively. For blue curves, the displayed color was RGB = (0, 0, 255), red curves RGB = (255, 0, 0), and green curves RGB = (0, 255, 0)

The displays used in this work to generate metameric spectra were a True HD-IPS liquid crystal display (LCD) on a LG G3 smartphone and a conventional cathode ray tube (CRT) monitor (Dell E770P). These displays use very different mechanisms to generate colors, which results in significantly different spectra when displaying the same color (i.e. metamers). The LCD display uses a backlight, typically a white LED, which is transmitted through color filter arrays (red, green and blue color filters) to produce its color response. Therefore, the emitted spectrum is the product of the LCD backlight and color filter transmission response. The CRT monitor uses an electron gun, and relies on a phosphorescent screen to control its spectrum in the visible wavelength range. Because the two display types use significantly different methods to generate colors, the two emission technologies have different spectral features for the individual red, green and blue color channels. The distinct features of the two displays are demonstrated in Figure S7(a), which shows the measured emitted spectrum of white light from each display (RGB = [255, 255, 255]). Figures S7(b, c) show the spectrum of each pure color channel (red, green and blue) for the LCD and CRT display, respectively.

Color accuracy of photographs:

In this work, we used digitally generated color samples from spectroscopic data to demonstrate the splitting of a metameric pair using an LCD and CRT monitor (Fig. 3). This method was used because, due to the difference in spectral response between a camera sensor and the human eye, it is difficult to obtain a precisely color-accurate photograph. This difficulty is shown in Figure S8, which shows the original photograph of the experimental setup in Fig. 3(c), an edited photograph that better approximates the actual color, and a digitally rendered color sample showing the “actual color”. The colors are rendered using CIE matching functions, as described above; the [X,Y,Z] values calculated using the matching functions and the measured spectrum can then be converted to the sRGB color space, which is the working color space

of most computers. Though the sRGB values will be the same across all display devices, the actual displayed color depends on the calibration of the monitor used. Therefore, the rendered color is Fig. S8(c) only represents the perceived color seen in the experiment when the monitor used to view the image has a perfect color calibration.

It is clear that Fig. S8(a) is significantly different than the generated color sample in Fig. S8(c), even though the camera used in S8(a) and the spectrometer used to acquire the sample that generated the color in S8(c) sampled the same light. Fig. S8(b) is the figure used in the main text (Fig. 3(b)), and was edited to more closely represent the color in S8(c) to prevent confusion. The rendered color samples using spectroscopic data represent the perceived colors of both monitors much more accurately.

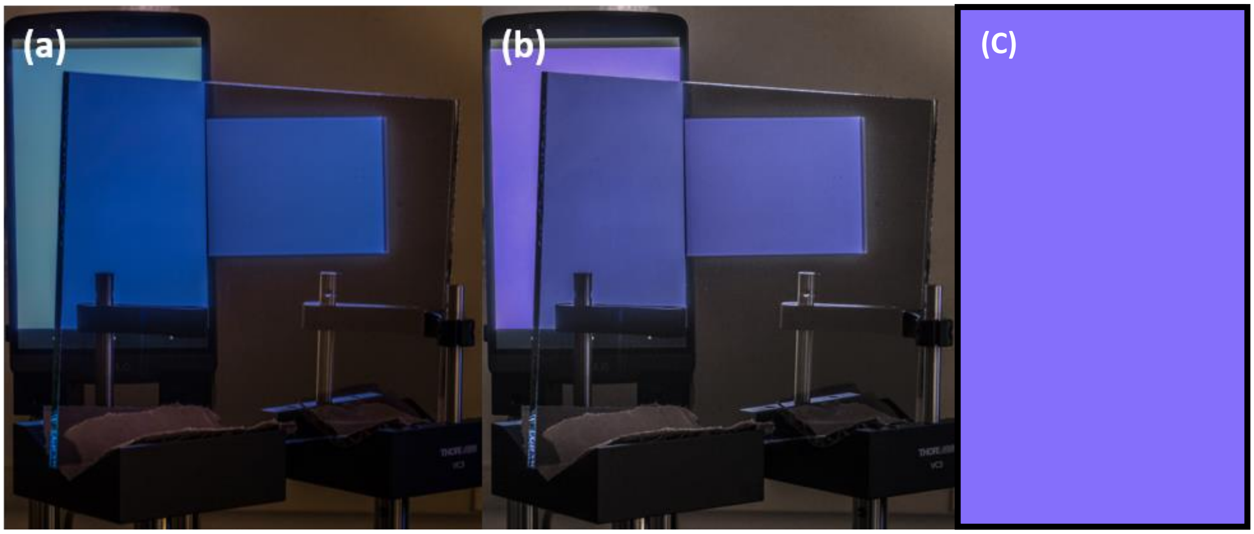

Figure S8: (a) Photograph of the setup shown in Fig. 3(c) of the main text, taken using a Sony α7R II camera. (b) The edited photograph that appears in Fig. 3(b) of the main text, which was modified to appear close in color to the rendered colors. This manipulation was performed to prevent confusion in the main text. (c) The actual color displayed during the experiment, rendered using spectra acquired using a grating spectrometer and cosine corrector.

<u>Hyperspectral images</u>

To demonstrate the utility of our wearable passive multispectral device in a more natural setting, we acquired a hyperspectral image of a complex scene, and applied the filters digitally (Fig. S9). The scene included a variety of blue and violet objects, including patches of color made using paints and pastels, plants, and a Morpho butterfly featuring a structural blue color [S5]. The image was obtained using a Middleton Spectral Vision MSV-500 High Sensitivity VNIR hyperspectral camera.

The enlarged images in Fig. S9(d) show the butterfly wing next to six similarly colored samples made using oil pastels, with no filters applied. In Fig. S9€ and Fig. S9(f), filters 2 and 1, respectively, are applied to these enlarged images. The numbers in each panel represent the CIE ΔE color difference between the oil pastel color and the butterfly wing, averaged over a small area to reduce pixel noise. Using filter 2 (Fig. S9(e)), the appearance of the butterfly wing becomes more dissimilar to the pastel samples compared to no filter (*i.e.*, the butterfly "blue" becomes easier to distinguish from the pastel "blue" using the filter). This again demonstrates the effect of partitioning the S cone to provide more spectral information. The improvement is absent for filter 1 (Fig. S9(f)), demonstrating the need for both filters in the design. We note that each filter creates a new set of metamers that may have been distinguishable before; by using two filters, the set of overlapping newly created metamers becomes significantly smaller. Therefore, as long as at least one filter creates an increase in contrast, more spectral information can be communicated to the visual system while decreasing the overall number of possible metamers. Although a slight yellow/green tint is applied to both filtered images, Fig. S9(b) and Fig. S9(c) have similar "white-balance" due to the white-balance condition enforced during the design process.

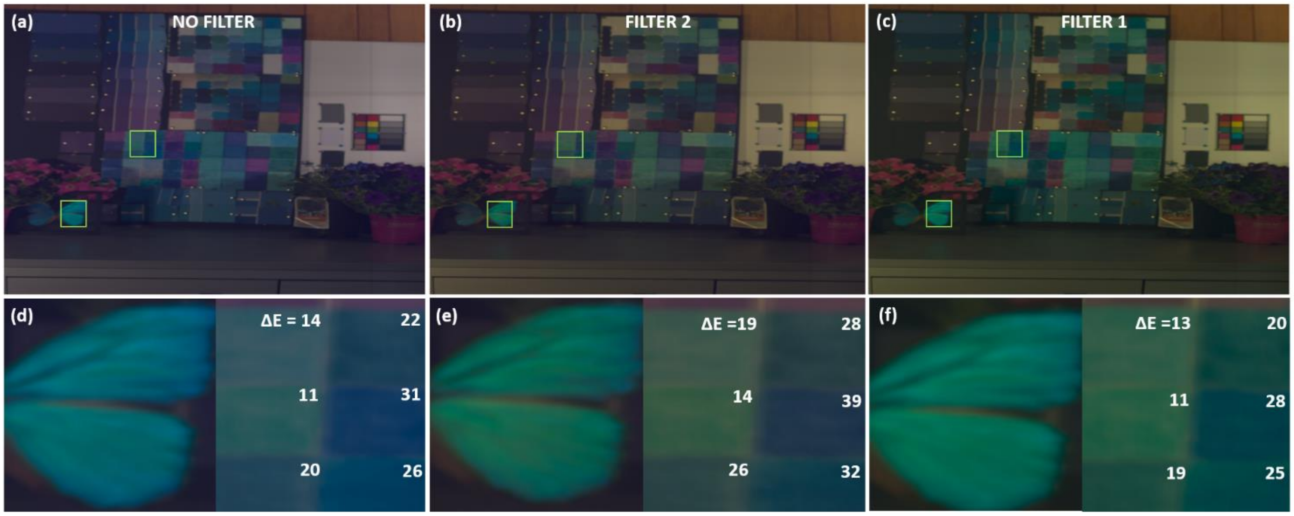

Fig. S9: (a) RGB rendering of a hyperspectral image with natural and artificially colored objects, with no filter applied. (b) The render with filter 2 applied, and (c) the render with filter 1 applied. (d) Magnified view of butterfly wing and paint samples (outlined in green in (a)). (e) and (f) are the same samples as in (d), with filters 2 and 1 applied, respectively. The numbers inside each paint sample are the ΔE color difference, rounded to the nearest integer, between the paint sample and the butterfly wing with each respective filter applied.

Supplementary References

S1. Giuseppe C, Ghaoui LE (2014) *Optimization Models* (Cambridge University Press).

S2. Boyd S, Vandenberghe L (2004) *Convex optimization* (Cambridge University Press).

S3. Grant M, Boyd S *CVX: Matlab software for disciplined convex programming*, version 2.1, http://cvxr.com/cvx, October 2016.

S4. Schmeder AW, McPherson DM (2014) Multi-band color vision filters and method by lp-optimization.

S5. P. Vukusic, J. R. Sambles, C. R. Lawrence, R. J. Wootton, Quantified interference and diffraction in single Morpho butterfly scales. *Proc. R. Soc. Lond. B Biol. Sci.* **266**, 1403–1411 (1999).
